# Introduction of Mutant GNAQ into Endothelial Cells Induces a Vascular Malformation Phenotype with Therapeutic Response to Imatinib

**DOI:** 10.3390/cancers14020413

**Published:** 2022-01-14

**Authors:** Maiko Sasaki, Yoonhee Jung, Paula North, Justin Elsey, Keith Choate, Michael Andrew Toussaint, Christina Huang, Rakan Radi, Adam J. Perricone, Victor G. Corces, Jack L. Arbiser

**Affiliations:** 1 Department of Dermatology, Emory University School of Medicine, Atlanta, GA 30322, USA; mpapke@emory.edu (M.S.); justin.elsey@emory.edu (J.E.); ckh5420@psu.edu (C.H.); rakan.h.radi@emory.edu (R.R.); 2 Departments of Dermatology, Veterans Affairs Medical Center, Decatur, GA 30322, USA; 3 Department of Human Genetics, Emory University School of Medicine, Atlanta, GA 30322, USA; yoonhee.jung@emory.edu (Y.J.); vgcorces@gmail.com (V.G.C.); 4 Department of Pathology, Laboratory Medicine Children’s Hospital of Wisconsin, Milwaukee, WI 53226, USA; pnorth@mcw.edu; 5 Departments of Dermatology, Pathology and Genetics, Yale University School of Medicine, New Haven, CT 06510, USA; keith.choate@yale.edu; 6 Department of Pathology, Emory University School of Medicine, Atlanta, GA 30322, USA; andy.toussaint@emory.edu (M.A.T.); adam.joseph.perricone@emory.edu (A.J.P.)

**Keywords:** GNAQ, vascular malformation, endothelial cells, Sturge Weber, vasculogenesis

## Abstract

**Simple Summary:**

Mutations in GNAQ underlie vascular malformations, including Sturge-Weber disease. In order to develop novel therapies for lesions with mutant GNAQ, we introduced mutant GNAQ into MS1 endothelial cells. Mutant GNAQ conferred a novel phenotype of progressive vascular malformations in mice. Chromatin analysis revealed upregulation of C-Kit in the vascular endothelial cells, and we found C-Kit to be highly expressed in Sturge-Weber disease. Given that imatinib is an FDA approved multikinase inhibitor that blocks C-Kit, we evaluated it in our mouse model, and showed that imatinib had activity against these vascular malformations. Repurposing imatinib should be evaluated in clinical trials, including Sturge-Weber disease.

**Abstract:**

GNAQ is mutated in vascular and melanocytic lesions, including vascular malformations and nevi. No in vivo model of GNAQ activation in endothelial cells has previously been described. We introduce mutant GNAQ into a murine endothelial cell line, MS1. The resultant transduced cells exhibit a novel phenotype in vivo, with extensive vasoformative endothelial cells forming aberrant lumens similar to those seen in vascular malformations. ATAC-seq analysis reveals activation of c-Kit in the novel vascular malformations. We demonstrate that c-Kit is expressed in authentic human Sturge–Weber vascular malformations, indicating a novel druggable target for Sturge–Weber syndrome. Since c-Kit is targeted by the FDA-approved drug imatinib, we tested the ability of imatinib on the phenotype of the vascular malformations in vivo. Imatinib treated vascular malformations are significantly smaller and have decreased supporting stromal cells surrounding the lumen. Imatinib may be useful in the treatment of human vascular malformations that express c-Kit, including Sturge–Weber syndrome.

## 1. Introduction

Sturge–Weber Syndrome (SWS) is a relatively common vascular malformation responsible for significant morbidity and mortality. These includes pain, seizures, glaucoma, facial deformity and mental retardation [1,2,3,4]. To date, there are no medical treatments that cause the regression of these lesions. SWS is caused by mutations of the GNAQ driver oncogene expressed in endothelial cells [5]. Similar GNAQ mutations are present in blue nevi and uveal melanoma, but there is little known malignant potential of SWS [6].

There are no known mouse models of vascular malformations caused by GNAQ mutation. The lack of mouse models has hindered the development of therapies for vascular malformations. In order to create a murine model of SWS, we used lentiviral introduction of mutant GNAQ into the murine microvascular MS1 cell line [7]. The resulting cells, MS1 GNAQ, form vascular malformations in mice that highly resemble those in human SWS. We used the Q209L mutation for the following reasons. First, it is the strongest activating mutation of the known GNAQ mutations. Second, it is mutated in both vascular and melanocytic lesions [8,9]. In order to determine potential targets induced by mutant GNAQ, we performed ATAC-seq to compare differences in chromatin accessibility between parental and GNAQ expressing lesions. Among the genes with increased chromatin accessibility, we discovered that c-Kit was elevated in the malformations expressing GNAQ. c-Kit (CD117) is a target of the FDA-approved drug imatinib, which targets c-Kit in addition to its original target Bcr-abl [10]. While imatinib inhibited growth of MS1 GNAQ Q209L lesions, c-Kit, the target of imatinib is expressed in human SWS tissue, indicating that c-Kit was an authentic and potential druggable target. Treatment of mice with imatinib resulted in significant growth inhibition of model vascular malformations in vivo. Repurposing the FDA-approved drug imatinib may be of use in the medical treatment of SWS.

## 2. Materials and Methods

### 2.1. Development of MS1 GNAQ Q209L Cells

Lentiviral particles were packaged in human embryonic kidney cells (HEK293T) cells using 1.25µg vector DNA, 1.25µg packaging plasmid psPAX2 (Addgene, Watertown, MA, USA) and 0.5µg envelope expressing plasmid pMD2.G (Addgene) per well in a 6-well plate with Lipofectamine 2000 (Thermo Fisher Scientific, Waltham, MA, USA) according to the manufacturer’s instructions [11]. MS1 cells were transduced using Polybrene (Millipore Sigma, Burlington, MA, USA) at a concentration of 5 µg/mL. The plasmid used was G49-GNAQ-Q209L which utilized the CMV promoter. Confirmation of expression of mutant GNAQ was accomplished by RT-PCR. RNA was isolated from the two tissues using the miRNEasy mini kit (Qiagen, Hilden, Germany) with on-column DNase treatment. RNA quality was assessed using Bioanalyzer (Agilent Technologies, Santa Clara, CA, USA) and Nanodrop (Thermo Fisher Scientific). cDNA was generated from 1 ug of RNA using the Superscript IV VILO with ezDNase enzyme (Thermo Fisher Scientific), according to manufacturer’s instructions. PCR was performed from the cDNA using primers specific for the GNAQ sequence that encompasses the Q209L sequence (GNAQ PCR F: GTGCTTAGAGTTCGAGTCCCCACCA and GNAQ PCR R: GTCTGGGTTCAGGTCCACGAACATC or GNAQ PCR F2: CTATCTTAATGACTTGGACCGCGTAGC and GNAQ PCR R: GTCTGGGTTCAGGTCCACGAACATC) using the Platinum SuperFi II PCR Master Mix (Thermo Fisher Scientific), according to manufacturer’s instructions. PCR products were purified using the QIAQuick PCR Purification kit (Qiagen) and purified PCR products were Sanger sequenced at Genewiz using the same primers that were used for PCR.

### 2.2. In Vivo Studies

MS1 and MS1GNAQ Q209L cell suspensions in growth medium were inoculated at 1x 10^6^ cells/mouse in the right flank of athymic Nu/Nu nude male mice (*n* = 5 per group) purchased from the Charles River Laboratories (Wilmington, MA, USA). 

### 2.3. Imatinib Treatment

Imatinib stock solution was prepared by dissolving 62.5 mg into 1 mL of sterile water and was further diluted to the prescribed final concentration in additional sterile water. Specifically, 100 uL of the stock solution was then added to 900 uL of sterile water for injection. Vehicle control (sterile water) or imatinib was administered intraperitoneally three times a week at 50 mg/kg/treatment. Imatinib treatment was initiated on the second day after the tumor cell injection, and the tumor volumes as well as the weight of the animals were recorded weekly thereafter [12].

### 2.4. RNA Extraction

Vascular malformation tissue was flash frozen in liquid nitrogen, then homogenized in QIAzol^®^ (Qiagen, Hilden, Germany) using a rotor-stator probe homogenizer until fully disrupted. RNA extraction was performed using Qiagen miRNeasy kit with on-column DNase treatment according to the manufacturer’s specifications (Qiagen). The concentration of RNA eluted in nuclease free water was determined using a Nano Drop 1000 (Thermo Fisher Scienticific). 1 μL of RNA was loaded and analyzed on Agilent 2100 Bioanalyzer (Agilent Technologies, Santa Clara, CA, USA), using RNA 6000 Nano assay for quality control prior to RNAseq. Total RNA was amplified and labeled using the Thermo Fisher Scientific Illuina™ Total Prep™ RNA Amplification kit (Thermo Fisher Scientific) according to the manufacturer’s protocol. Labeled cRNA was hybridized to Illumina HT12 bead array according to manufacturer instruction. 

### 2.5. Assay for Transposase-Accessible Chromatin Using Sequencing (ATAC-Seq)

After cells were counted, the nuclei from 50,000 cells were isolated with lysis buffer (10 mM Tris-HCl pH 7.4, 10 mM NaCl, 3 mM MgCl2) containing 0.1% NP40, 0.1% Tween-20, and 0.01% digitonin. The purified nuclei pellet was then resuspended in the transposase reaction mix containing 0.05% digitonin and incubated for 30 min at 37 °C. Following incubation, cells were treated with proteinase K at 55 °C for 2 h and gDNA was isolated by phenol:chloroform:isoamyl alcohol and EtOH precipitation. Library amplification was done with 2x KAPA HiFi mix (Kapa Biosystems, Wilmington, MA, USA) and 1.25 µM indexed primers using the following PCR conditions: 72 °C for 5 min; 98 °C for 30 s; and 10–11 cycles at 98 °C for 10 s, 63 °C for 30 s, and 72 °C for 1 min.

All libraries were sequenced using Illumina NovaSeq 6000 sequencers (Illumina, San Diego, CA, USA) and 50 bp paired-end format as previously described [13,14,15]. MACS2 was used for peak calling of subnucleosomal reads.

### 2.6. Immunohistochemistry

Formalin-fixed paraffin embedded Sturge–Weber tissue was obtained from the Pediatric Pathology Department (Boston Children’s Hospital of Wisconsin, Milwaukee) following institutional review board approved protocol. These sections were cut at 4µm thickness, deparaffinized, and blocked with peroxidase block and serum-free protein block (Dako, Agilent Technologies). The slides were incubated with the primary, etv-2 (Abcam, Cambridge, United Kingdom #ab181847), c-Kit antibody (Dako), followed by secondary antibody. We tried using the DMAB-DCC056 antibody from Creative Diagnostics (New York, NY, USA) for GNAQ Q209L, but it did not work in our hands.

### 2.7. Statistical Analysis

The statistical analysis of tumor volumes was performed as previously described; all the statistical analyses were performed using Microsoft Office Excel (Microsoft Corporation, Richmond, WA, USA) [3]. Briefly, tumor volume was calculated using the formula volume = (L × W^2^) × 0.52, where L (length) was defined to be the longer dimension of the tumor. Five replicates were in each group, and unpaired two-tailed Student’s t-test was performed to determine the significant difference between the two groups, with significance determined at *p* < 0.05.

Analysis of significance of peaks of chromatin accessibility was determined using 2-fold change and *p*-value <= 0.001 for calling differential peaks. A list of fold change and *p*-value with the gene list is in Table 1.

## 3. Results

### 3.1. Expression of GNAQ Q209L in MS1 Cells Results in Vascular Malformations in Mice

**Figure 1:**
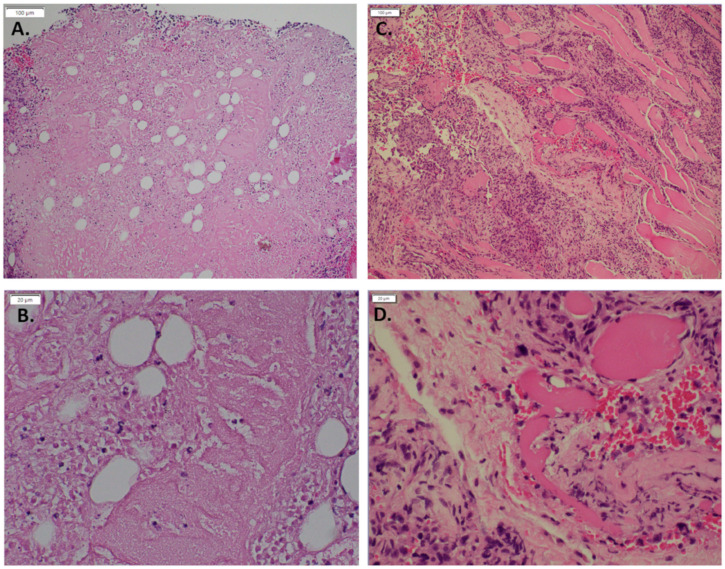
Hematoxylin and eosin of MS 1 control in vivo compared to MS1 GNAQ Q209L (**A**). Control (10×) (**B**). Control (40×) (**C**). GNAQ Q209L (10×) (**D**). GNAQ Q209L (40×) Note the increased size of lumens in cells expressing GNAQ Q209L versus the solid pattern of cells in the MS1 parental lesions.

To demonstrate whether expression of mutant GNAQ in endothelial cells results in recapitulation of vascular malformations in mice, we injected MS1 cells expressing GNAQ Q209L and their parental controls into nude mice. MS1 GNAQ Q209L cells formed vascular malformations with enlarged lumens, while parental MS1 cells formed highly cellular solid lesions (Figure 1A,B). Both MS1 and MS1 GNAQ Q209L cells express the master endothelial regulatory transcription factor Etv2 [16,17]. 

### 3.2. Expression of GNAQ Q209L Results in Alterations in Chromatin Accessibility

**Figure 2:**
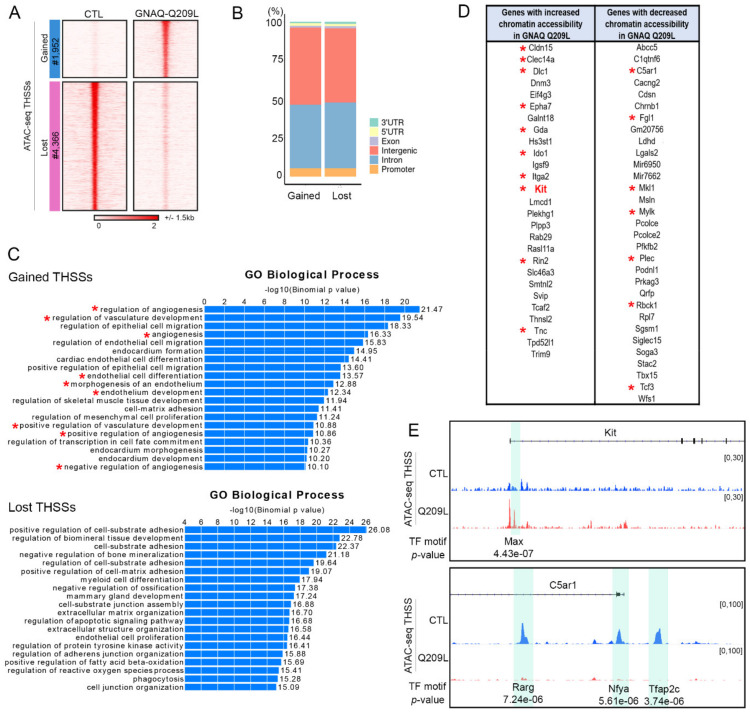
(**A**). Genomic sites with altered chromatin accessibility (THSSs) in vascular malformations of mice injected with MS1 cells expressing GNAQ-Q209L (GNAQ-Q209L) or not (CTL). (**B**). Distribution of differential ATAC-seq peaks with respect to gene features. (**C**). Gene ontology biological process enrichment analysis of genes containing differential ATAC-seq peaks. Asterisk denotes pathways involved in angiogenesis or vasculogenesis. (**D**). List of genes with differential chromatin accessibility; Asterisk denotes genes involved in angiogenesis or vasculogenesis. (**E**). Examples of differential ATAC-seq peaks in two genes related to angiogenesis.

To gain insights into the mechanisms by which MS1 GNAQ Q209L cells cause vascular malformations in mice, we analyze possible changes in chromatin structure in cells of the vascular malformations induced by injection of MS1 cells expressing GNAQ Q209L using ATAC-seq. We find that malformations caused by expression of GNAQ Q209L show a gain of 1952 and loss of 4366 transposase hypersensitive sites (THSSs) with respect to samples from mice injected with MS1 cells, suggesting a dramatic alteration in the binding of transcription factors (Figure 2A). Sites gained and lost are present at promoters but more frequently are located in introns and intergenic regions, suggesting they correspond to enhancer sequences (Figure 2B). Gene ontology analyses reveal that most gained sites are located in genes related to angiogenic regulation, vascular development, and endothelial cell differentiation (Figure 2C). Many of the genes with increased or decreased chromatin accessibility at the promoter have been previously identified as positive or negative regulators of angiogenesis and/or vasculogenesis (Figure 2D). For example, c-Kit shows a dramatic increase in chromatin accessibility at the promoter region as well as at a putative enhancer located in the first intron containing an E-box binding site for the transcription factors Myc-Max. The effect on c-Kit was highly significant (differential log2FC = 3.95, *p*-value = 0.000000136). (Figure 2E). Of the genes that were differentially regulated, we chose to study c-Kit further given that it is the only gene on the list that can be targeted by a drug which is already FDA-approved.

On the other hand, C5ar1, mutation of which has been shown to increase wound healing in mice [18], shows decreases of chromatin accessibility at the promoter and at two putative enhancers normally bound by Retinoic Acid Receptor gamma (Rarg), Nfya, and Tfap2c (Figure 2E). 

### 3.3. Imatinib Inhibits Growth of Sturge–Weber Model Vascular Malformations In Vivo

**Figure 3:**
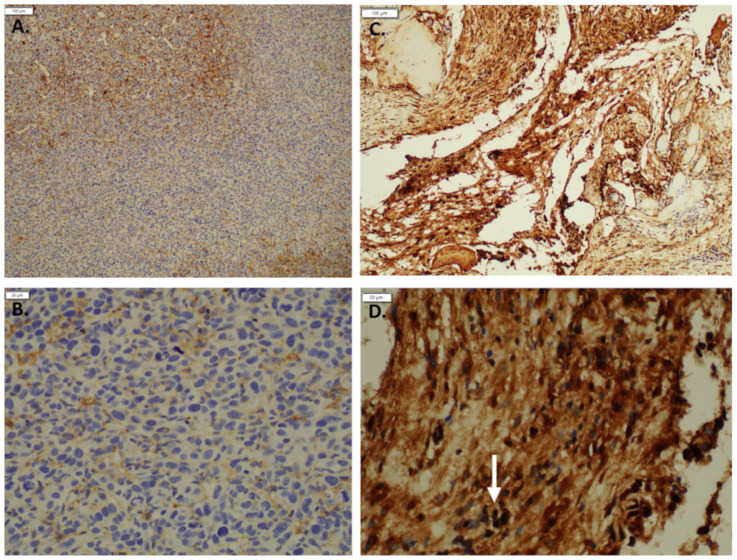
Immunohistochemistry staining: c-Kit staining in (**A**). MS 1 control (10×) (**B**). MS1 control (40×) compared to (**C**). MS1 GNAQ Q209L (10×) (**D**). MS1 GNAQ Q209L with the white arrow pointing out cells strongly stained positive for c-Kit (40×). The symbol * denotes genes or pathways involved in angiogenesis.

**Figure 4:**
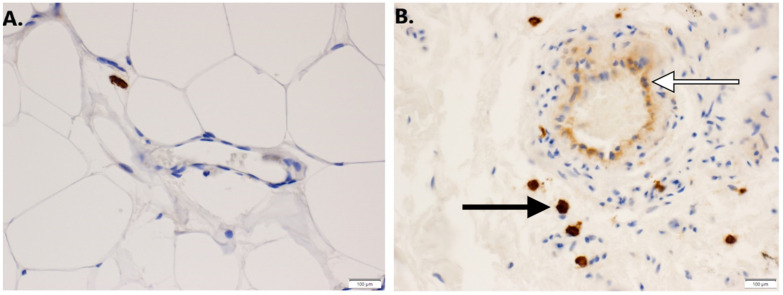
(**A**). c-Kit staining in control dermal endothelial cells from normal skin. (10×). (**B**). c-Kit positive staining in endothelial cells (white arrow) and perivascular mast cells (black arrow) in human SWS tissue. (10×).

**Figure 5:**
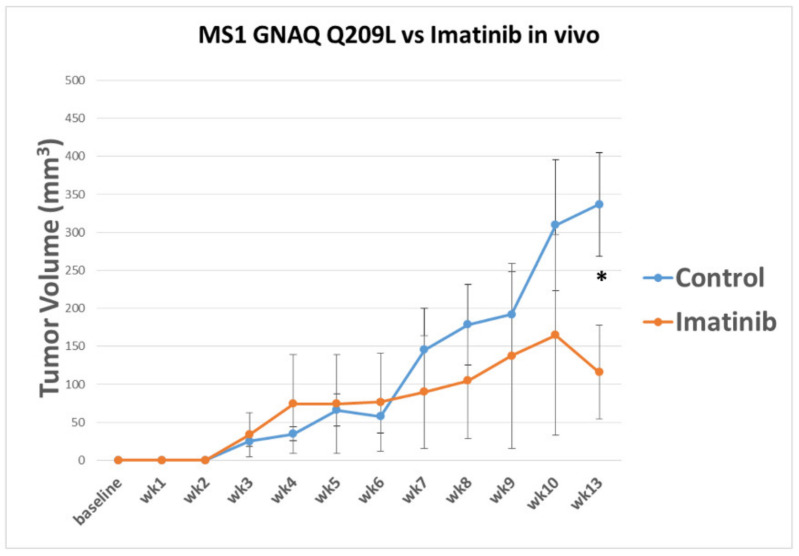
Imatinib treatment results in reduced tumor volume over 13 weeks. Mice were inoculated with MS1 GNAQ Q209L (1 × 10^6^ cells) and then treated with imatinib via IP administration three times a week at a concentration of 50 mg/kg. Tumor growth was compared to control vehicle (sterile water). Results indicate a possible reduction in tumor growth over period of observance. Asterisk denotes significance (*N* = 5, *p*-value = 0.04, * *p* ≤ 0.05).

**Figure 6:**
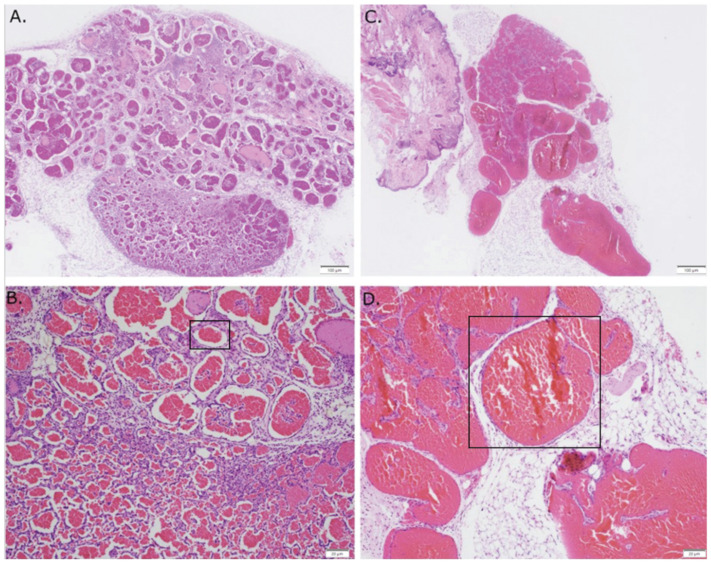
Hematoxylin and eosin of MS1 GNAQ Q209L in vivo compared against imatinib treatment. (**A**). Control (10×) (**B**). Control (40×) (**C**). Imatinib treatment (10×) (**D**). Imatinib treatment (40×). Note the increased size of lumens in the lesions from mice treated with imatinib. The blood vessels in the imatinib treated mice demonstrate a relative paucity of stromal supporting cells compared with the vehicle controls.

The increase in chromatin accessibility observed around the promoter of the c-Kit gene, which is the receptor for stem cell factor (SCF), suggests that its expression may be altered in cells of vascular malformations. We confirmed the induction of c-Kit by mutant GNAQ by demonstrating low expression in the parental MS1 cells to high level expression in MS1 cells transduced with mutant GNAQ (Figure 3). To examine whether c-Kit is associated with Sturge–Weber syndrome, we examined its expression in human SWS tissue. We find that both endothelial and mast cells express c-Kit (Figure 4). Since c-Kit is inhibited by imatinib, which also inhibits the bcr-abl kinase and platelet derived growth factor receptor beta (PDGFRb) [19], we treated mice with GNAQ Q209L-induced vascular malformations with imatinib and noted that imatinib treatment diminished progressive growth of SWS vascular malformations (Figure 5). We examined vehicle and imatinib treated lesions histologically; imatinib treated lesions showed decreased amounts of stromal tissue in respect to vascular lumens (Figure 6).

## 4. Discussion

Vascular malformations are common abnormalities of endothelial cells. Unlike the more common hemangiomas of infancy, vascular malformations do not regress, but grow over time. Their continued growth can endanger vital structures such as the brain and eye, and often result in disfigurement, requiring multiple surgeries. Pain is also a common symptom of SWS. Vascular malformations are broadly classified as lymphatic and hemogenic in origin, and they have significant differences. First, driver oncogenic mutations are more common in hemogenic vascular malformations (GNAQ, PI3 kinase, tie-2, Braf, and ras) than in lymphatic vascular lesions [20,21,22,23,24]. Second, lymphatic vascular malformations are more responsive to systemic mTORC1 inhibition than hemogenic vascular malformations [25,26,27].

While antiangiogenic therapies have been tried against hemogenic vascular malformations (rapamycin, propranolol, avastin), none are highly successful against hemogenic vascular malformations [28,29] The inability to culture cells from these lesions long term, due to oncogene-induced senescence [30], has provided a significant barrier to studying these cells in vitro and in vivo. Furthermore, introduction of dominant oncogenes into primary endothelial cells is fraught with the same issues of oncogene-induced senescence. Transgenic approaches in introducing mutant GNAQ into endothelial cells have so far been unsuccessful. 

In order to overcome these issues, we have used the MS1 cell line, a well-characterized murine endothelial cell that encodes a temperature sensitive large T antigen [7]. The large T antigen is active at a low level at 37 °C, allowing the cells to override oncogene-induced senescence and thus expansion for in vitro and in vivo use. Endothelial cells expressing GNAQ were noted to have a distinct phenotype in vivo. While MS1 derived endothelial cells formed lesions with very small lumens, introduction of GNAQ resulted in increased numbers of large lumen vessels.

Limitations of this study include the fact that we used GNAQ Q209L instead of R183, which is the most common GNAQ mutation. However, we validated that c-Kit, which is a druggable target in the Q209L lesions, is also present in authentic human SWS tissue as well. c-Kit is the receptor for stem cell factor and is a target of the multikinase inhibitor, imatinib. While imatinib is not specific for c-Kit, inhibiting other kinases may increase the success of imatinib in patients with SWS. This is the first time, to our knowledge, that c-Kit has been identified as a potential target in human SWS. Of interest, c-Kit is expressed in venous malformations and progenitor cells in lymphatic vascular malformations, which suggest that c-Kit inhibition may be useful in the treatment of additional vascular malformations [31,32]. Introduction of GNAQ Q209L induces striking histological changes in vivo, notably extensive vasoformation. Interestingly, abnormal vessels in human vascular malformations also exhibit a prominent stromal element [33]. Treatment of mice harboring GNAQ Q209L induced vascular malformations with imatinib decreased the size of these lesions, but led to larger lumens which appeared to have a less prominent stromal component. Our observations suggest that stromal cells exhibit a supportive role in maintaining abnormal vessels in SWS, and treatment with imatinib results in vessels with larger lumens but smaller lesions. This suggests that imatinib, an FDA-approved drug, might be useful in the treatment of SWS. Smaller lesions might be able to undergo embolization and surgical removal. In addition, imatinib treated vascular malformations may be more susceptible to pharmacologic interventions, such as propranolol and sirolimus, which as single agents are ineffective against SWS. Our cells will also provide the research community a valuable resource to test both novel and repurposed drugs for SWS. Finally, our studies provide a rationale for clinical trials of imatinib for SWS.

## 5. Conclusions

Overall, the presented findings demonstrate that introducing the GNAQ Q209L mu-tation into MS1 cells leads to the growth of vascular malformations in mouse models closely resembling the lesions found in patients with SWS. Furthermore, these lesions over-expressed c-Kit (as evidenced by chromatin analysis and immunohistochemical staining), which is also elevated in human SWS legions. Treatment with imatinib reduced lesion volume in MS1 GNAQ Q209L mouse models, indicating the potential for this FDA-approved drug as a treatment for SWS. Furthermore, the development of a murine model for the vascular malformations found in SWS can facilitate research on treatments for these lesions.

## Figures and Tables

**Table 1:** List of genes with chromatin accessibility induced at least 2 fold by introduction of GNAQ and level of significance.

Gene	Log2(FC)	*p*-Value
Cldn15	2.9	2.10 × 10^−11^
Clec14a	4.53	2.85 × 10^−06^
Dlc1	4.05	3.80 × 10^−07^
Dnm3	4.43	8.97 × 10^−09^
Eif4g3	3.86	0.0000381
Epha7	4.54	2.74 × 10^−09^
Galnt18	4.53	1.00 × 10^−05^
Gda	2.52	5.69 × 10^−07^
Hs3st1	3.83	7.23 × 10^−09^
Ido1	2.68	4.58 × 10^−05^
Igsf9	2.89	6.50 × 10^−09^
Itga2	2.54	2.48 × 10^−06^
Kit	3.95	1.36 × 10^−07^
Lmcd1	5.64	2.48 × 10^−09^
Plekhg1	5.24	1.42 × 10^−07^
Plpp3	3.53	2.41 × 10^−10^
Rab29	2.53	1.37 × 10^−08^
Rasl11a	4.03	7.50 × 10^−17^
Rin2	5.73	0.0000106
Slc46a3	1.79	5.86 × 10^−08^
Smtnl2	4.31	1.42 × 10^−06^
Svip	2.89	2.01 × 10^−08^
Tcaf2	2.8	3.17 × 10^−10^
Thnsl2	4.4	1.29 × 10^−08^
Tnc	6.12	5.03 × 10^−14^
Tpd52l1	2.88	3.94 × 10^−06^
Trim9	4.17	4.42 × 10^−05^

## Data Availability

The data presented in this study are available in this article.
